# Human CD3+ T-Cells with the Anti-ERBB2 Chimeric Antigen Receptor Exhibit Efficient Targeting and Induce Apoptosis in ERBB2 Overexpressing Breast Cancer Cells

**DOI:** 10.3390/ijms18091797

**Published:** 2017-09-08

**Authors:** Rusheni Munisvaradass, Suresh Kumar, Chandramohan Govindasamy, Khalid S. Alnumair, Pooi Ling Mok

**Affiliations:** 1 Department of Biomedical Science, Faculty of Medicine and Health Sciences, Universiti Putra Malaysia, 43400 UPM Serdang, Selangor, Malaysia; rusheni91@gmail.com; 2 Genetics and Regenerative Medicine Research Center, Universiti Putra Malaysia, 43400 UPM Serdang, Selangor, Malaysia; sureshkudsc@gmail.com; 3 Department of Medical Microbiology and Parasitology, Universiti Putra Malaysia, 43400 UPM Serdang, Selangor, Malaysia; 4 Department of Community Health Sciences, College of Applied Medical Sciences, King Saud University, Riyadh 11433, Saudi Arabia; gcmohanphd@gmail.com (C.G.); khalidsalnumair@gmail.com (K.S.A.)

**Keywords:** breast cancer, chimeric antigen receptor (CAR), human epidermal growth factor receptor (ERBB2), immunotherapy, transduction, human T-cells

## Abstract

Breast cancer is a common malignancy among women. The innate and adaptive immune responses failed to be activated owing to immune modulation in the tumour microenvironment. Decades of scientific study links the overexpression of human epidermal growth factor receptor 2 (ERBB2) antigen with aggressive tumours. The Chimeric Antigen Receptor (CAR) coding for specific tumour-associated antigens could initiate intrinsic T-cell signalling, inducing T-cell activation, and cytotoxic activity without the need for major histocompatibility complex recognition. This renders CAR as a potentially universal immunotherapeutic option. Herein, we aimed to establish CAR in CD3+ T-cells, isolated from human peripheral blood mononucleated cells that could subsequently target and induce apoptosis in the ERBB2 overexpressing human breast cancer cell line, SKBR3. Constructed CAR was inserted into a lentiviral plasmid containing a green fluorescent protein tag and produced as lentiviral particles that were used to transduce activated T-cells. Transduced CAR-T cells were then primed with SKBR3 cells to evaluate their functionality. Results showed increased apoptosis in SKBR3 cells co-cultured with CAR-T cells compared to the control (non–transduced T-cells). This study demonstrates that CAR introduction helps overcome the innate limitations of native T-cells leading to cancer cell apoptosis. We recommend future studies should focus on in vivo cytotoxicity of CAR-T cells against ERBB2 expressing tumours.

## 1. Introduction

Cancer has become an endemic disease that is particularly prevalent in the modern world. Of all cancers, breast cancer is often seen to occur at an alarming rate in women [1,2]. In the United States, approximately 5–10% of initial breast cancer diagnosis indicates metastatic breast cancer (MBC), which has increased relapse and mortality rates [3]. The occurrence of cancer is commonly linked to genetic code malfunctions, leading to abnormal expression of human epidermal growth factor receptor 2 (ERBB2), progesterone [4], oestrogen receptors [5], tumour protein P53 [6], and others.

Overexpression of the ERBB2 protein and its receptor on the surface of cancer cells are highly correlated to MBC occurrence. ERBB2 (also known as HER2 or neu) belongs to the epidermal growth factor receptor (EGFR) [7] family. ERBB2 amplification and overexpression have been shown to possess a prevalence of 15–25% in breast cancer patients [8]. This overexpression, in turn, lowers the efficiency of chemotherapy and endocrine therapy, resulting in an earlier relapse with poor prognosis. ERBB2 is also found to be overexpressed in ovarian [9], stomach [10], bladder [11], salivary, and lung carcinomas [12]. Current conventional therapy to target ERBB2 antigen in breast cancer includes treatment with trastuzumab, a humanized monoclonal antibody that binds to the extracellular domain of the receptor [13]. Trastuzumab inhibits mitogen-activated protein kinases and phosphatidylinositol-4, 5-bisphosphate 3-kinase/protein kinase B pathways, leading to the suppression of cell growth and proliferation, triggering of ERBB2 degradation through tyrosine kinase-ubiquitin ligase activity, and attraction of immune cells to ERBB2 overexpressing tumour sites through antibody-dependent cellular cytotoxicity (ADCC) [14]. However, the efficiency of this targeted therapy is shown to be only 12–34% [15,16].

Alternative treatment strategy of manipulating immune cells such as T lymphocytes in adoptive cellular therapy (ACT) could efficiently target cancer cells, resulting in 50% clinical regression [17]. However, the existing challenge in ACT is that the identified tumour antigens are mostly self-derived and, thus, poorly immunogenic as they could adopt several mechanisms to render themselves immune resistant to T-cells [18]. Hence, various methods to increase specificity in cancer cell targeting of tumour-associated antigens (TAAs) have been developed. Chimeric antigen receptor (CAR) represents one of the most advanced and highly-researched gene recombination technologies to be incorporated in ACT. A CAR links an antigen-specific single-chain antibody fragment (scFv) to intracellular signalling domains (cluster of differentiation 28 molecule, CD28 and T-cell receptor CD3 zeta chain, CD3-ζ) of the T-cell receptor (TCR) [19]. The binding event of the TAA-specific scFv to its specific TAA could, therefore, trigger desired T-cell activation and effector functions. Therefore, T-cells modified with CAR are capable of directly triggering an immune response in a non-major histocompatibility complex (MHC)-restricted manner [20]. This is advantageous and superior to other therapies as tumour cells could evade the immune system by downregulating the MHC recognition complex [21]. As ERBB2 is a surface antigen that is overexpressed in breast cancer, a CAR designed to target ERBB2 could be the ideal solution for the treatment of breast cancer.

Breast cancer targeting using CAR technology has been well documented with studies reporting the generation of CAR for breast cancer-associated tumour antigens such as carbonic anhydrase IX [22], ERBB2 [23], mesothelin [24], EGFR variant III (EGFRvIII) [25], carcinoembryonic antigen (CEA) [26], and Mucin-1 (MUC-1) [27]. In the present study, we aimed to establish genetically-modified T-cells expressing CAR that is specific for the ERBB2 antigen. In contrast to other studies that used CD4+ or CD8+ T-cells, we directly transduced the *CAR* gene into CD3+ cells. We successfully showed that these genetically modified CD3+ cells were able to specifically target and induce apoptosis in the ERBB2 overexpressing breast cancer cell line, SKBR3. We also discussed the advantages of transduction into CD3+ versus CD4+ or CD8+ cells from the perspective of tumouricidal efficiency for clinical applications.

## 2. Results

### 2.1. Successful Transduction of Lentiviral Particles Encoding Chimeric Antigen Receptor (CAR) into Human CD3+ T-Cells

**Figure 1:**
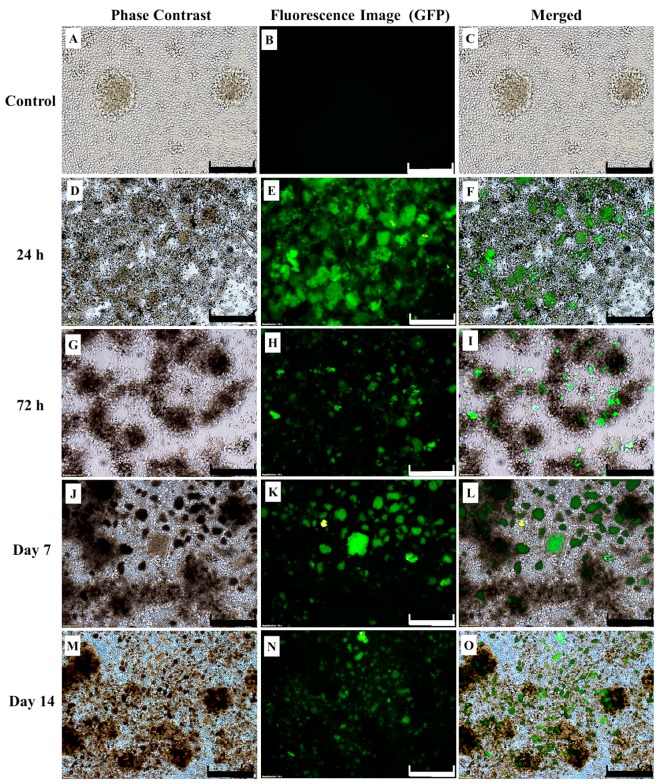
Verification of transduction efficiency of the chimeric antigen receptor (CAR) based on fluorescence microscopy of green fluorescent protein (GFP) expression on human CD3+ T-cells. Phase contrast, GFP fluorescence, and merged images of human T-cells are shown. The images shown are control (**A**–**C**), 24 h (**D**–**F**), 72 h (**G**–**I**), day 7 (**J**–**L**), and day 14 (**M**–**O**) post-transduction by spinoculation (day 0). (**A**–**C**) Clumped T-cells are observed in due to activation by DynaBeads prior to transduction. T-cells that have successfully undergone transduction (CAR-T cells) showed significant amounts of GFP expression at 24 h (**D**–**F**) and 72 h (**G**–**I**). These images are compared to the control, non-transduced T-cells (**A**–**C**). Cells were imaged at 100× magnification (the scale bar represents 100 μm).

**Figure 2:**
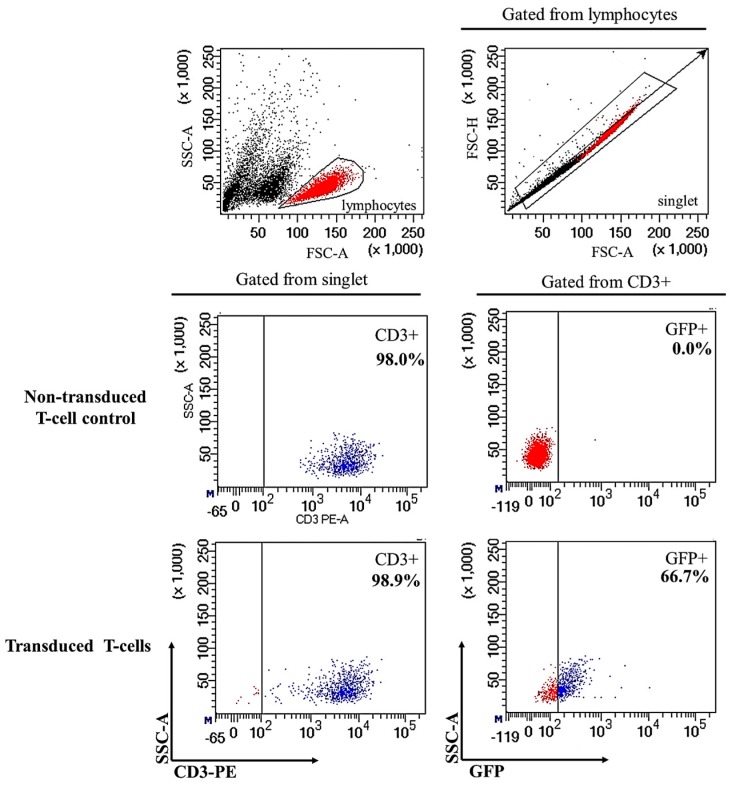
Verification of the transduction efficiency based on flow cytometric analysis of CAR-transduced human T-cells (CAR-T cells). The transduction efficiency was evaluated by the percentage of GFP-positive T-cells 72 h post-transduction. The cell population was gated at lymphocytes. Singlet was gated from the lymphocyte population to remove residual cell clumps following disaggregation and eliminate auto-fluorescence. Subsequently, CD3+ cells were gated from the singlet population, and GFP+ cells were gated from the CD3+ population. The GFP positive cells indicate that 66.7% of T-cells were successfully transduced with *CAR*-encoding lentiviral particles, generating CAR-T cells. CD3 was stained by fluorochrome phycoerythrin (PE). For each antibody, isotype-matched mouse immunoglobulin γ antibody was used as the isotype control.

The lentivirus was packaged by 293FT cells once the presence of *CAR* gene within the lentiviral expression transfer plasmid was confirmed (Figure S1). Successful lentiviral production was indicated by the green fluorescence expressed by 293FT cells (Figure S1) and the viral supernatant was used to transduce human CD3+ T-cells. The effective isolation and activation for expansion of human CD3+ T-cells were shown in supplementary data. Human CD3+ T-cells were purified from peripheral blood mononuclear cells (PBMC) (Figure S2) and culture-expanded with DynaBeads Human T-activator CD3/CD28 and interleukin 2 (IL-2) before transduction via spinoculation (Figure S3). Following spinoculation, fluorescence microscopic examination of the transduced cells showed the majority of cells expressing green fluorescent protein (GFP) at a high intensity at 24 h post-transduction (Figure 1D–F). However, GFP expression decreased at 72 h post-transduction (Figure 1G–I). Flow cytometric analysis at 72 h revealed GFP expression by approximately 66.7% of the CD3+ T-cells (Figure 2). Following that, GFP expression did not decrease with prolonged culture and was seen for up to 14 days, indicating both successful transduction and stable *CAR* gene integration into CD3+ T-cells (Figure 1M–O). Successfully transduced CD3+ T-cells are termed CAR-T cells. In contrast, GFP signals were not detected in non-transduced T-cells by both fluorescence microscopy (Figure 1A–C) and flow cytometry analysis (Figure 2).

### 2.2. CAR-T Cells Showed Presence of Cell Surface Activation Markers upon Co-Culture with SKBR3 Cells

**Figure 3:**
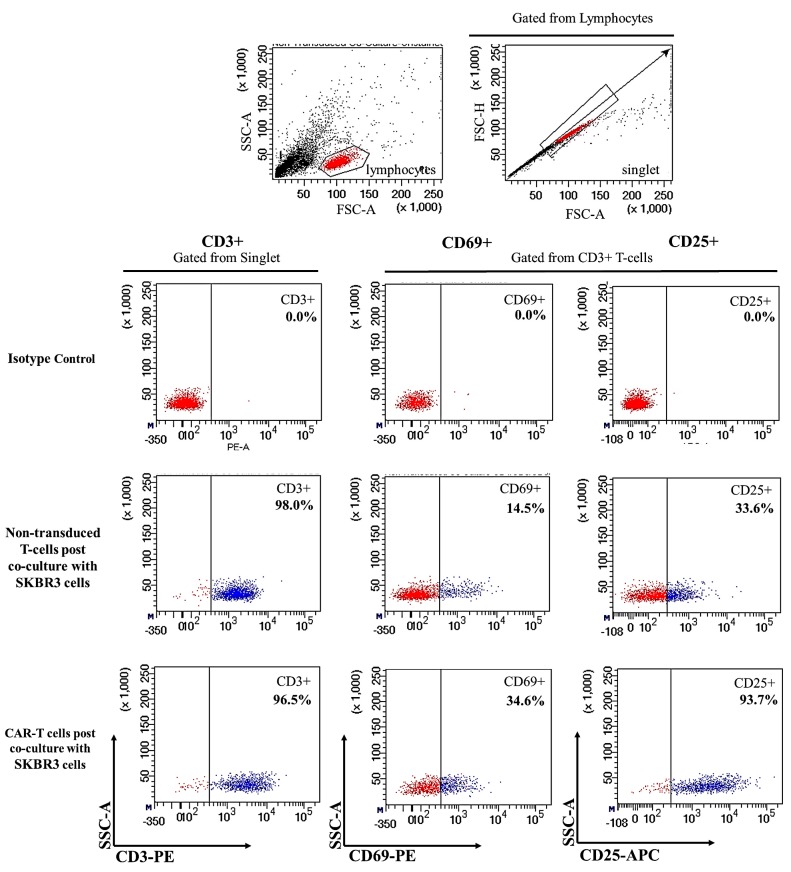
Flow cytometric detection of activation cell surface markers, CD69 and CD25 after co-culture with SKBR3 cells. The cell population was gated at lymphocytes. Singlet was gated from the lymphocyte population to remove residual cell clumps following disaggregation and eliminate auto-fluorescence. Subsequently, CD3+ cells were gated from the singlet population, and CD69+ and CD25+ cells were gated from the CD3+ population. CAR-T cells, 72 h post co-culture with SKBR3 cells, showed significantly higher levels of activation markers (CD69+: 34.6%, CD25+: 93.7%) than those by non-transduced T-cells (control) 72 h post co-culture with SKBR3 cells (CD69+: 14.5%, CD25+: 33.6%). CD3+ percentages were consistently high at 98.0% and 96.5% for non-transduced and CAR-T cells, respectively, 72 h post co-culture with SKBR3 cells. The isotype control used were activated T-cells that were not co-cultured with SKBR3 cells. CD3 was stained by fluorochrome PE, CD69 by PE, and CD25 by allophycocyanin (APC) fluorochromes. For each antibody, isotype-matched mouse immunoglobulin γ antibody was used as the isotype control.

Produced CAR-T cells were co-cultured with the SKBR3 breast cancer cell line for 72 h to test its activation and functionality. Flow cytometric analysis was employed to validate the activation of CAR-T cells upon co-culture. The results showed a high number of CD3+ surface markers (96.5%) in CAR-T cells that corresponded to 98.0% CD3+ surface markers detected in non-transduced T-cells (Figure 3). The early activation CD69 cell surface marker increases significantly in CAR-T cells compared to non-transduced T-cells (34.6% vs. 14.5%). Similarly, the late activation cell surface marker, CD25, was also increased significantly in CAR-T cells compared to non-transduced T-cells (93.7% vs. 33.6%) upon co-culture with SKBR3 cells, as shown in Figure 3. Both CAR-T cells and non-transduced T-cells displayed higher CD69 and CD25 activation marker expression compared to DynaBeads stimulated background activation of 9.2% and 9.0% (Figure S3), respectively.

### 2.3. CAR-T Cells Effectively Secreted IFN-γ and Subsequently Lysed the SKBR3 Cells 

**Figure 4:**
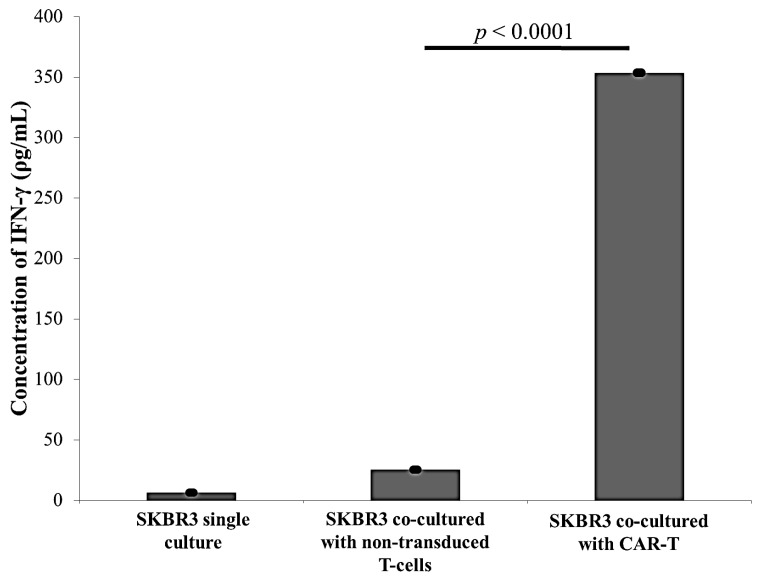
Detection of interferon-γ production upon co-culture of CAR-T cells with SKBR3 cells. The figure shows interferon-gamma (IFN-γ) secretion in the supernatant of the T-cells and ERBB2 overexpressing cancer cell line, SKBR3, after 72 h of co-culture. Only 6.42 pg/mL IFN-γ was produced in the SKBR3 single culture supernatants (*n* = 6). SKBR3 co-cultured with non-transduced T-cells showed 25.57 pg/mL IFN-γ production (*n* = 6). SKBR3 co-cultured with CAR-T showed IFN-γ at concentrations of 353.63 ± 10.64 pg/mL was produced in the supernatant of this experimental group with a significant *p* value of *p* < 0.0001 (*n* = 6) compared to that of SKBR3 co-cultured with non-transduced T-cells. The X-axis indicates the experimental groups, while the Y-axis indicates the concentration of the IFN-γ (pg/mL) produced with the scale bar up to 400 pg/mL. The resulting data was reported as a bar chart of the experimental mean ± standard error of the mean (S.E.M.) (*n* = 6). The *p* < 0.0001 was determined with respect to SKBR3 co-cultured with CAR-T compared to non-transduced T-cells by ordinary one-way analysis of variance (ANOVA).

Following co-culture of CAR-T cells and non-transduced T-cells with SKBR3, and a single culture of SKBR3 (negative control), human interferon gamma (IFN-γ) levels within supernatants were determined using a sandwich ELISA kit (BioLegend, San Diego, CA, USA). The amount of IFN-γ secreted in CAR-T cells co-cultured with SKBR3 cells were significantly higher by almost 14-fold compared to that by non-transduced T-cells or SKBR3 single culture (Figure 4).

**Figure 5:**
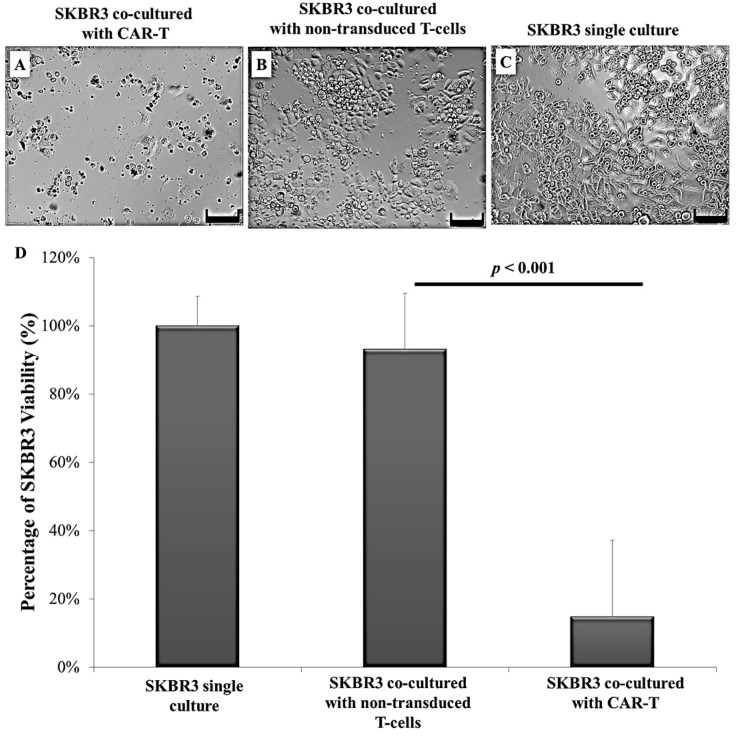
Detection of efficiency of CAR-T cells in SKBR3 cell lysis. The effectiveness of CAR-T cells and non-transduced T-cells in the lysis of SKBR3 cells was observed by phase contrast microscopy and measured by the CellTiter 96 Cell Proliferation Assay (MTS). (**A**) Phase contrast image of SKBR3 co-cultured with CAR-T cells. The image showed a minimal amount of attached SKBR3 cells on the plate, indicating low cell viability; (**B**) phase contrast image of SKBR3 co-cultured with non-transduced T-cells. The image showed high numbers of attached SKBR3 cells on the plate, indicating high cell viability; (**C**) phase contrast image of SKBR3 single culture. The image showed high numbers of attached SKBR3 cells indicating high cell viability. Cells were visualized by inverted light microscopy. Cells were imaged at 100× magnification (the scale bar represents 200 μm); (**D**) the graph indicates that cell viability of SKBR3 cells co-cultured with CAR-T (*n* = 7) was extremely low compared to that of SKBR3 cells co-cultured with non-transduced T-cells (*n* = 6) or SKBR3 single culture cells (*n* = 6). Statistical analysis showed an extremely low *p* value of <0.001 for co-culture with CAR-T cells compared to non-transduced T-cells or SKBR3 single culture. The percentage of viable SKBR3 cells that remained attached to the plate was only 14.84% in CAR-T co-cultured with SKBR3 cells compared to 93.2% in SKBR3 cells co-cultured with the non-transduced T-cell. The SKBR3 single culture was observed to show 100% cell viability. The X-axis indicates the experimental groups while Y-axis indicates the percentage of SKBR3 cell viability. MTS data was reported as a bar chart of the experimental mean ± standard error of the mean (S.E.M) with *p* < 0.001 with respect to the experimental SKBR3 cell viability upon co-culture with CAR-T cells compared to non-transduced T-cells by ordinary one-way ANOVA analysis.

When observed by microscopy, only a few SKBR3 cells were viable and remained attached to the plate upon co-culture with CAR-T cells. Meanwhile, a high number of SKBR3 cells were still viable and remained attached to the plate upon co-culture with non-transduced T-cells. The SKBR3 single culture was maintained as a negative control. Results indicated that SKBR3 cell death was higher upon co-culture with CAR-T cells (Figure 5A–C). The effectiveness of CAR-T cells and non-transduced T-cells in the lysis of SKBR3 tumour cells was measured by the CellTiter 96 Cell Proliferation Assay (MTS) (Promega, Madison, WI, USA) and Fluorescein Isothiocyanate (FITC) Annexin V Apoptosis Detection Kit (Becton Dickinson (BD), Franklin Lakes, NJ, USA). MTS assay results showed that SKBR3 cell viability was only 14.84% upon co-culture with CAR-T cells and was significantly lower than the viabilities of SKBR3 cells co-cultured with non-transduced T-cells (93.2%, *p* < 0.001) or single culture of SKBR3 (100%, *p* < 0.001) (Figure 5D).

**Figure 6:**
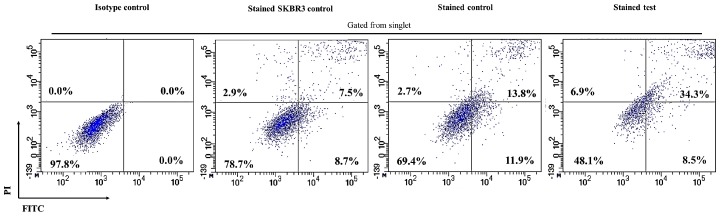
Detection of efficiency of CAR-T cells in SKBR3 cell lysis by FITC Annexin V Apoptosis Detection Kit. The FITC Annexin V Apoptosis Detection Kit was used to measure the amount of apoptosis seen in SKBR3 cells following co-culture with CAR-T cells (stained test) and non-transduced T-cells (stained control). SKBR3 single culture (stained SKBR3 control) was designated as the background. The cell population was gated at SKBR3 cells. Singlet was gated from the SKBR3 cells population to remove residual cell clumps following disaggregation and eliminate auto-fluorescence. Subsequently, apoptotic cells were gated from the singlet population. Viable cells in the apoptosis assay are stained FITC−/Propidium iodide− (PI), necrotic cells are stained FITC−/PI+, early apoptotic cells are stained FITC+/PI−, while late apoptotic cells are stained FITC+/PI+. Upon co-culture with CAR-T cells, SKBR3 cells showed increased late apoptotic cells and necrotic cells compared to non-transduced T-cells. Meanwhile, the number of viable SKBR3 cells decreased in co-culture with CAR-T compared to non-transduced T-cells. In comparison, stained SKBR3 control showed lower late apoptotic and necrotic cells. The data is a representative of one technical replicate from cells (*n* = 6) that were pooled together prior to flow cytometric analysis.

In addition, 8.5% of SKBR3 cells, co-cultured with CAR-T cells, showed early apoptosis compared to 11.9% of SKBR3 cells after co-culture with non-transduced T-cells, indicating no changes in early apoptosis (Figure 6). However, a marked increase in late apoptotic SKBR3 cells were observed in the co-culture with CAR-T cells (34.3%) compared to non-transduced T-cells (13.8%) (Figure 6). The number of viable SKBR3 cells decreased considerably upon co-culture with CAR-T cells compared to non-transduced T-cells (48.1% vs. 69.4%), while an increase in necrotic SKBR3 cells were seen (6.9% vs. 2.7%). Meanwhile, 8.7% and 7.5% of the SKBR3 single culture, which served as the control, were early and late apoptotic cells, respectively (Figure 6).

## 3. Discussion

The ERBB2 cell surface antigen is an excellent target in CAR-T technology as the extracellular domain of the receptor could easily be subjected to the antibody binding mechanism [28] and is currently used as a potential target in cancer vaccine trials [29,30]. However, ERBB2 is an antigen found in low quantities within native healthy cells such as lung [8] and gut cells [31]. Precautionary measures must be implemented while targeting ERBB2 [32] in order to reduce the possibility of off-target reactivity. To reduce the possibility of chronic cytotoxicity by CAR-T cells, we did not include the extra T-cell co-stimulatory 4-1BB intracellular domain in the CAR sequence, commonly used in the third generation of the CAR molecule that further enhances CAR-T cell survivability and cytokine secretion. Serious adverse events, such as cytokine storm and death, have been observed in a clinical trial following adoptive transfer of third generation CAR-T [33]. Morgan et al. [23] transduced third generation CAR into peripheral blood lymphocytes by the γ-retroviral system and co-cultured the transduced cells with ERBB2-positive cancer cell lines, demonstrating impressive CAR-T cell apoptotic activity. However, the authors reported massive T-cell activation and clinical respiratory failure triggered by the physiological levels of ERBB2 expressed on normal lung epithelium. These effects would be less likely to be observed in second generation CAR-T cells targeting ERBB2 [34] as the lack of 4-1BB motif ensured no excessive T-cell proliferation and activation either for short- or long-term. Thus, a second generation CAR with a less aggressive, but therapeutically enhanced function seems to be the better choice.

In the present study, we manipulated CD3+ T-cells for the delivery of *CAR* since a heterogeneous effector cell population of CD8+ and CD4+ [35] could execute cytolysis more efficiently than a single population of CD8+ or CD4+ CAR-T cells [35,36]. Human CD8+ T-cells predominantly use two pathways in cytolysis execution, the perforin and granzyme B exocytosis and, to some extent, death receptor signalling via Fas/Fas-ligand [37] or tumour necrosis factor/tumour necrosis factor-receptor [38]. Meanwhile, human CD4+ T-cells execute effector functions by principally secreting cytokines such as IFN-γ, lymphotoxin α [39], and IL-2 [40], that activate and recruit cytolytic immune cells such as CD8+ T-cells, natural killer cells, and macrophages [41]. However, studies have shown that CD4+ CAR-T cells can rapidly lyse their targets as efficiently as CD8+ CAR-T cells in the short-term in vitro assays. Mediation of CD4+ CAR-T cell cytolysis may be executed by the granzyme/perforin pathway [35,36,42]. Indication of a synergistic effect in CAR-T cells from both subsets of CD8+ and CD4+ T-cells have also been observed in human immunodeficiency virus (HIV) CAR trials [43]. Sommermeyer et al. [35] demonstrated that upon antigen binding, CD4+ CAR T-cells proliferated more effectively and produced abundant Th1 cytokines (e.g., IFN-γ and IL-2) that contributed to the proliferation, survival, and efficacy of CD8+ CAR-T-cells, in addition to perforin and granzyme secretion. Similar synergistic activity of CD4+ and CD8+ CAR-T cells has also been observed in murine models [44]. Moreover, PBMC consists of almost 70% CD3+ T-cells compared to only 21% of CD8+ T-cells [45,46]. Thus, adopting the strategy of transducing CAR into CD3+ T-cells could save both time and cost of cell preparation.

The aim of this study is to establish ERBB2 specific CAR-T cells that could specifically target and induce apoptosis in breast cancer cell line, SKBR3. Thus, we constructed a second generation CAR (ERBB2 scFv-CD8α-CD28-CD3-ζ) [1,47] (Figure S1) into lentiviral particles that were used to transduce activated CD3+ T-cells via spinoculation. We observed high transduction efficiency (66.7%) (Figure 2) and stable CAR integration into CD3+ T-cells, as shown by constitutive GFP expression by T-cells up to day 14 (Figure 1M–O). Subsequent co-culture results of the generated CAR-T cells with SKBR3 cancer cell line indicated strong activation in CAR-T cells, which was measured by the high expression of CD69 and CD25 (Figure 3). In addition, increased production of the cytokine IFN-γ by CD4+ Th1 helper cells and CD8+ cytotoxic T-cells, once antigen-specific immunity develops [48], can also be used as a parameter to measure T-cell functionality. Our findings indicate that MHC-independent antigen (ERBB2) recognition by CAR-T cells enabled them to perform efficient cytolysis of target cells and secrete significant amounts of IFN-γ [16,23,28] (Figure 4). This indicates successful ligation of ERBB2 scFv antibody on the CAR to the antigen and activation of intracellular signalling transduction in CD3+ T-cells to produce pro-inflammatory IFN-γ [16,49]. To optimally detect CAR-T cells cytotoxicity in inducing apoptosis of SKBR3 cells via the granzyme and perforin pathway, a flow cytometric-based apoptosis assay was used. The results obtained showed that CAR-T cells, not non-transduced T-cells, proved to be able to induce significant cell death in SKBR3 cancer cells after 72 h of co-culture via both viability and apoptosis assays [16,50] (Figure 5 and Figure 6).

This study could be significant in targeting not only ERBB2 positive breast cancer due to CAR-T specificity for ERBB2, but also a myriad of other cancers [9,10,11,12] that express the ERBB2 antigen. Several studies demonstrated that, unlike haematological malignancies [19,27,51], solid tumours are more difficult to be targeted by CAR-T cells because of specific tumour characteristics, limited tumour specific antigens, and the resilient immunosuppressive tumour microenvironment [52]. Limited efficiency coupled with off target adverse effects [22,23] have been seen in CAR-T cells targeting solid tumours, including renal carcinoma [22], neuroblastoma [53], ovarian cancer [54], and even ERBB2 positive breast cancer [23]. However, Ahmed et al. [55] showed that ERBB2-specific CAR-T cells were able to persist without causing any toxic effects in a phase I/II clinical study, proving that targeting of solid tumours with CAR-T cells are feasible.

To improve the efficiency of CAR-T immunotherapy for cancer cell killing, dual CAR systems including tandem CARs that target two different tumour antigens [27] on a single tumour cell can be designed. Examples of other TAAs that could be used in combination with ERBB2 in targeting breast cancer include melanoma-associated antigen-A4 (MAGE-A4) [56], mesothelin [57], and MUC-1 [27]. Meanwhile, off-target toxicity [23] of CAR-T cells can be reduced by introducing suicide genes [57], such as *herpes-simplex-thymidine-kinase* and *inducible-caspase-9* [58]. On the other hand, the emerging CRISPR-associated RNA-guided endonuclease Cas9 [59] may be exploited to disrupt the programmed cell death protein-1 on primary human T-cells from cancer patients, which is responsible for immunosuppression.

This study is significant in demonstrating that strategies employed to transduce CAR into human T-cells at CD3+ levels could give rise to effective ERBB2 antigen-specific CAR-T cell cytotoxic activity, and reduce the time and cost of CAR-T cell preparation [45,46]. CAR-T cells could overcome trastuzumab resistance and target tumours that express ERBB2 at moderate levels (e.g., osteosarcoma, glioblastoma, and medulloblastoma) that commonly escape trastuzumab treatment [60]. The fourth generation CAR (TRUCK CAR) which redirects T-cells by secreting IL-12, complementing and enhancing CAR-T antitumor activity, could also be adopted as a universal therapeutic mechanism [61]. Future studies aim to focus on in vitro experimentation across multiple cell lines with differing magnitude of ERBB2 expressions to evaluate the CAR-T cells affinity in successfully inducing cytotoxic activity [62]. In addition, in vivo testing against ERBB2 overexpressing breast tumours must be conducted to ensure safety profile and efficient CAR-T cells homing towards tumour site [61]. Such data will ensure the identification of suitable CAR-T cells dosage that will induce cancer cell death without harming non-cancerous cells with physiological levels of ERBB2 in patients during treatments. CAR-T for ERBB2 cancer cell targeting may be used to complement other treatment modalities such as immune checkpoint inhibitors [63], oncolytic virus [64], and nanoparticles [65], or even with other types of immune effector cells, such as natural killer cells [66].

## 4. Materials and Methods

### 4.1. Collection, Isolation, and Processing of Human T-Cells

Whole blood samples (10 mL each) were collected in ethylenediaminetetraacetic acid (EDTA) tubes from healthy human donors at the National Blood Centre, Malaysia after obtaining written informed consent. All studies were performed in accordance to the International Harmonization Good Clinical Practice Guidelines approved by the Medical Research and Ethics Committee, National Institute of Health, Ministry of Health, Malaysia (ethical approval ID: NMRR-14-587-20201, 9 October 2014). The diluted blood samples were processed to isolate human peripheral blood mononuclear cells (PBMC) by the Ficoll-Paque Plus (1.077 g/mL; Amersham Biosciences, Amersham, UK) density gradient separation method. Cells were enumerated by the trypan blue exclusion method using a haemocytometer.

### 4.2. Isolation of Human CD3+ T-Cells from Peripheral Blood Mononuclear Cells (PBMC)

The CD3+ T-cells were further isolated using the EasySep™ Human T Cell Enrichment Kit (Stem Cell technologies, Vancouver, BC, Canada). T-cell suspension was prepared in phosphate-buffered saline (PBS) within a 5 mL polystyrene tube (Becton Dickinson (BD), Franklin Lakes, NJ, USA) at a density of 5 × 10^7^ cells/mL. EasySep™ Human T Cell Enrichment Cocktail and EasySep™ D Magnetic Particles was added at 50 μL/mL cells, and the cells were incubated and placed into a magnet for cell separation according to the manufacturer’s protocol. The negatively selected CD3+ T-cell population were cultured.

### 4.3. Culture, Expansion, and Characterisation of Human T-Cells and the SKBR3 Cell Line

Isolated T-cells were cultured in a 25 cm^2^ plastic flask containing advanced Roswell park memorial institute (RPMI) 1640 media (Thermo Fisher Scientific, Waltham, MA, USA), supplemented with 10% foetal bovine serum (Thermo Fisher Scientific) and 1% penicillin/streptomycin (Thermo Fisher Scientific), at a density of 1 × 10^6^ cells/mL at 37 °C in a humidified 5% CO_2_ atmosphere with daily monitoring. The cells were also supplemented with DynaBeads Human T-Activator CD3/CD28 (Thermo Fisher Scientific) at a 1:10 beads to cell ratio and 50 U/mL of interleukin 2 (IL-2) (PeproTech, Rocky Hill, NJ, USA) in order to induce T-cell activation and expansion. SK-BR-3 (SKBR3) breast cancer cell line (ATCC HTB-30, Manassas, VA, USA) were cultured in a 25 cm^2^ plastic flask containing advanced RPMI 1640 media, 10% foetal bovine serum, and 1% penicillin/streptomycin at a seeding density of 1 × 10^5^ cells/mL at 37 °C in a humidified 5% CO_2_ atmosphere. Once the cells reached confluence, they were detached using 0.25% trypsin-EDTA (Thermo Fisher Scientific) and sub-cultured into new flasks at a similar cell density for maintaining culture.

### 4.4. Immunophenotyping of Human T-Cells

To detect surface antigens, aliquots of cultured T-cells were centrifuged at 1000× *g* for 5 min to pellet the cells, which were then washed twice with PBS (pH 7.2). The cells were then suspended in PBS and incubated with fluorescein isothiocyanate (FITC), allophycocyanin (APC), phycoerythrin (PE), or phycoerythrin cyanine7 (PE-Cy7)-conjugated monoclonal antibodies on ice for 45 min. The antibodies used were CD3, CD25, and CD69 (Affymetrix eBioscience Inc., Santa Clara, CA, USA). After incubation, the cells were washed twice with PBS by centrifugation at 300× *g* for 5 min and subjected to flow cytometric analysis. Unstained, fluorochrome-matched and non-specific isotype-labelled cells were used as controls and run in parallel to the experiment. The stained samples were assessed using BD FACS Canto II (Becton Dickinson (BD), Franklin Lakes, NJ, USA). Gating at FACS acquisition was applied to exclude any dead cells and cell debris. Ten thousand events were acquired, and the data from stained cells were analysed using FACSDiva 6.1.3 software (Becton Dickinson (BD), Franklin Lakes, NJ, USA).

### 4.5. Generation of Lentiviral Plasmid Coding for CAR

pReceiver-Lv 183 lentiviral expression transfer plasmid, encoding the CAR and downstream green fluorescent protein (GFP), was purchased from GeneCopoeia (Rockville, MD, USA). To this lentiviral expression plasmid, the 1515 base-pair long CAR, from patent WO2012031744 (National Center for Biotechnology Information (NCBI) accession number: JB399738, 2 Oct 2013), was sub-cloned by GeneCopoeia. CAR is made of domains of an antigen specific (ERBB2) single-chain antibody fragment (scFv) (anti-ERBB2 scFv) connected to the hinge region CD8α, transmembrane, and intracellular CD28 and CD3-ζ chains of TCR. The presence of CAR was confirmed by amplification of the bacterial transformants of the pReceiver-Lv 183 lentiviral expression transfer plasmid by polymerase chain reaction.

### 4.6. Lentiviral Production and Transduction to Produce CAR-Expressing Human T-Cells (CAR-T Cells)

The Expression Arrest-In Trans-Lentiviral Packaging Mix kit (30 μL) (Thermo Fisher Scientific) was used in combination with the lentiviral expression plasmid (pReceiver-Lv 183) (42 μg) containing CAR and Lipofectamine 2000 (36 μL) (Thermo Fisher Scientific) to produce complete recombinant viral particles. Lentivirus was produced using human embryonic kidney 293FT cells, a packaging cell line (Thermo Fisher Scientific) with incubation at 37 °C in a humidified 5% CO_2_ atmosphere for 12 h. After replacement with fresh culture medium, the transfected 293FT cells had grown to confluence and exhibited green fluorescence in their cytoplasm when observed under a fluorescence microscope (Olympus IX51, Olympus, Tokyo, Japan).

The lentiviral-containing culture medium was collected in sterile capped tubes, 48 and 72 h post-transfection. The tubes were then centrifuged at 500× *g* for 10 min to discard the cell debris. Following centrifugation, the supernatant is filtered through a polyvinylidene fluoride (Merck Millipore, Billerica, MA, USA) low-protein-binding filter with a pore size of 0.45 μm. The supernatant is then ultracentrifuged at 107,000× *g* for 2.5 h at 4 °C using the Optima L-100 XP Ultracentrifuge of SW41 rotor (Beckman Coulter, Brea, CA, USA). The obtained pellet was suspended in 300 μL of advanced RPMI media, supplemented with 10% foetal bovine serum, and incubated for 18 h with gentle shaking at 4 °C. The suspended pellet is used immediately for T-cell transduction thereafter.

Human T-cells (2 × 10^5^ cells/mL) were inoculated into a 24-well plate containing advanced RPMI media, with 10% foetal bovine serum (Thermo Fisher Scientific), supplemented with DynaBeads Human T-activator CD3/CD28, and 50 U/mL of IL-2 in triplicates, 18 h prior to viral transduction. The virus suspension (300 μL) and polybrene (Sigma-Aldrich, St. Louis, MO, USA) were added to the T-cells at a final concentration of 8 μg/mL. For non-transduced T-cells, RPMI media was added with polybrene instead of the virus suspension. The human T-cells (2 × 10^5^ cells/mL) were spinoculated at 800× *g* for 1.5 h at 32 °C. The centrifuged mixture was harvested and inoculated into 24-well plates, which were placed in a 37 °C incubator with humidified 5% CO_2_ atmosphere. The medium was replaced with fresh complete medium after 12 h of incubation. Cell transduction efficiency was measured by detecting the GFP expression with a fluorescence microscope (Olympus IX51, Olympus, Tokyo, Japan) and BD FACS Canto II.

### 4.7. Co-Culture of CAR-T Cells with SKBR3 Cells and T-Cell Activation Marker Detection

SKBR3 cells (1 × 10^4^ cells/mL) were added to a 96-well plate 18 h prior to co-culture. CAR-T (1 × 10^5^ cells/mL) were inoculated into each well with SKBR3 cells to achieve a ratio of one effector cell to 10 target cells (1:10) of SKBR3 cells to CAR-T cells (*n* = 6). Non-transduced T-cells (1 × 10^5^ cells/mL) co-cultured with SKBR3 cells (*n* = 6) served as the control. Single culture of SKBR3 (1 × 10^4^ cells/mL) served as the negative control (*n* = 6). Following 72 h of co-culture, the CAR-T and non-transduced T-cells (1 × 10^5^ cells/mL) were collected for flow cytometric analysis for the presence of the T-cell activation marker, CD69 andCD25.

### 4.8. Determination of IFN-γ Expression by Enzyme-Linked Immunosorbent Assay (ELISA)

IFN-γ secretion was measured by the IFN-γ ELISA assay (BioLegend, San Diego, CA, USA) as per the manufacturer’s protocol. Secreted proteins in the supernatants of CAR-T cells co-cultured with SKBR3 (*n* = 6), non-transduced T-cells co-cultured with SKBR3 (*n* = 6), and SKBR3 single culture (*n* = 6) were measured after 72 h using a microplate reader (Molecular Devices, Sunnyvale, CA, USA) at 450 nm. A correction wavelength of 570 nm was used to correct for optical imperfections. The corrected optical density (OD) obtained from the background control containing only the culture medium was subtracted from the corrected OD of the samples.

### 4.9. Effect of CAR-T Cells on Survivability of SKBR3 Cells In Vitro Following Co-Culture

Following 72 h of co-culture, the proportion of viable SKBR3 cells were tested using CellTiter 96 AQueous One Solution Cell Proliferation Assay (MTS) (Promega, Madison, WI, USA) after co-culture with either CAR-T cells or non-transduced T-cells. MTS reagent (300 µg/mL) were added directly into the cell culture media (200 μL) after removal of T-cells, and the cells are incubated for 1 h and 45 min at 37 °C in a humidified 5% CO_2_ atmosphere. After the incubation period, the amount of formazan product formed at the absorbance value of 490 nm was recorded using a colorimetric microplate reader (Molecular Devices). The percentage of cell viability was calculated by normalizing the mean absorbance value of SKBR3 co-cultured with CAR-T cells (*n* = 7) with SKBR3 co-cultured with non-transduced T-cells (*n* = 6), multiplied by 100%.

Meanwhile, apoptosis of SKBR3 cells were detected using the PE Annexin V Apoptosis Detection Kit I (BD) (catalogue number: 559763). Briefly, the cells were washed with PBS and incubated with PE Annexin V in the dark for 15 min at 25 °C and analysed using flow cytometry.

### 4.10. Statistical Analysis

Quantitative data were presented as mean values ± standard error of the mean (*n* = 6). Statistical analysis of IFN-γ secretion and cell viability was performed using GraphPad Prism (Version 6.0; GraphPad Software, San Diego, CA, USA) with a one-way ANOVA test and *p* < 0.05 were considered statistically significant. Normality of distribution and equal variance of data were confirmed using the Kolmogorov-Smirnov test with Lilliefors and Levene’s tests, respectively, *p* < 0.05 was considered statistically significant.

## 5. Conclusions

In conclusion, we have successfully demonstrated transduction of *CAR* into human CD3+ T-cells. The transduced T-cells were activated and exhibited significant cytotoxicity on ERBB2 expressing breast cancer cell line. This may overcome the multitude of limitations inherent to ACT, such as heterogeneity of effector cell populations and poorly-defined target specificity [16], while maintaining its advantages of high intrinsic cytotoxic potential [67] and ability to home in on tumour sites [49].

## Supplementary Materials

Supplementary materials can be found at www.mdpi.com/1422-0067/18/9/1797/s1.
