# Supplementary material for: Human CD3+ T-Cells with the Anti-ERBB2 Chimeric Antigen Receptor Exhibit Efficient Targeting and Induce Apoptosis in ERBB2 Overexpressing Breast Cancer Cells
Source: Int J Mol Sci. 2017 Sep 8;18(9):1797. doi: 10.3390/ijms18091797 (PMC5618474; doi:10.3390/ijms18091797)
Supplement: Supplementary file 1 [file ijms-18-01797-s001.pdf]

International Journal of *Molecular Sciences*

MDPI

Human CD3+ T-cells with the Anti-ERBB2 Chimeric Antigen Receptor Exhibit Efficient Targeting and Induce Apoptosis in ERBB2 Overexpressing Breast Cancer Cells

Rusheni Munisvaradass, Suresh Kumar, Chandramohan Govindasamy, Khalid S. Alnumair and Pooi Ling Mok *

1.0. Human CD3+ T-cells effectively isolated and activated for expansion

Human T-cells in the PBMC which were isolated using density gradient centrifugation, were found to be only 68.3% positive for the T-cell specific surface marker, CD3 by flow cytometric analysis. Magnetic separation of CD3+ T-cells from PBMC could enhance the purity of CD3+ cells up to 99.5%, characterized by the distinct population shift to the right seen on the Pe-Cy 7 channel (Figure S2). The purified CD3+ T-cells were cultured in vitro supplemented with activation reagents, DynaBeads Human T-activator CD3/CD28 and IL-2. Morphological observations showed individual spherical CD3+ suspension T-cells formed clumps, indicating active cell proliferation. After a 72 h stimulation with DynaBeads Human T-activator CD3/C28 and IL-2, flow cytometric analysis revealed positive detection of early CD69 and late CD25 T-cell activation surface markers. The findings showed that 9.2% and 9.0% of the CD3+ cells expressed CD69 and CD25, respectively, compared to non-activated T-cells (Figure S3). The positive CD69 and CD25 cell surface marker expression seen is indicative of the background activation in CD3+ T-cells stimulated by the culture media supplement, DynaBeads Human T-activator CD3/CD28 and IL-2. The results suggest successful culture-expansion of T-cells.

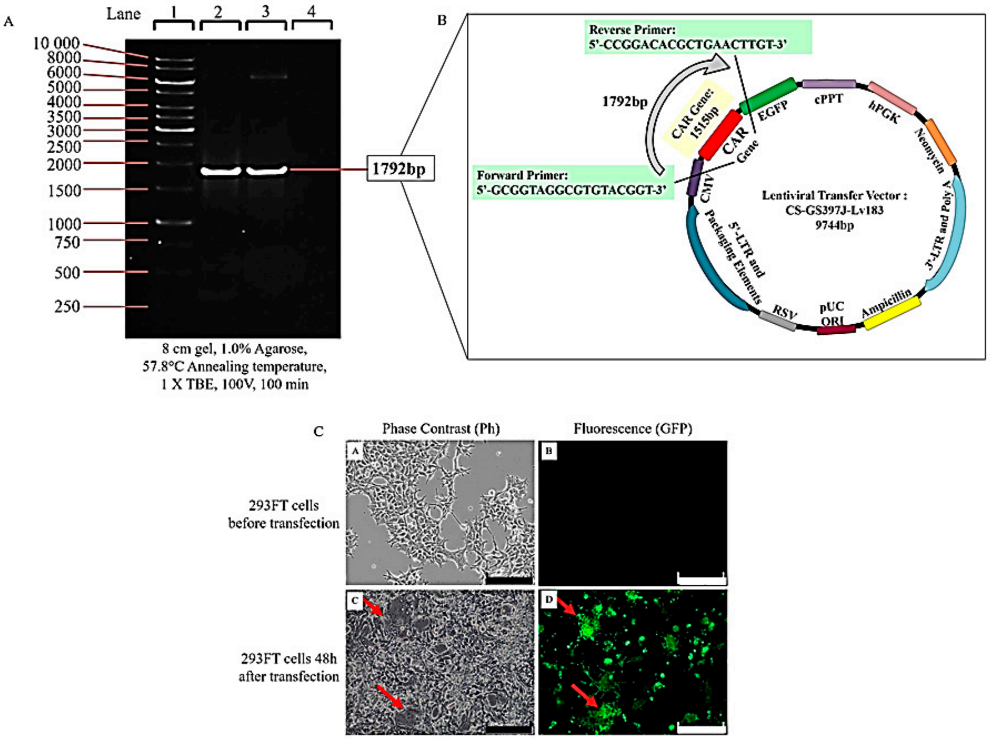

Figure S1. Schematic representation of the chimeric antigen receptor (CAR) encoding lentiviral transfer vector (CS-GS397J-Lv183) construct and lentiviral transfection of human kidney 293FT cells. (A)

International Journal of *Molecular Sciences*

MDPI

Confirmation of *CAR* from CS-GS397J-Lv183 transfer plasmid. Resulting plasmid DNA was processed in 1.0% agarose gel in the following lanes: lane 1 (1 kb base pair DNA ladder), lane 2 (positive control containing 97.3 ng/µl of CS-GS397J-Lv183 CAR-encoding lentiviral plasmid), lane 3 (maxiprep purified sample containing 97.3 ng/µl of transformed CAR-encoding lentiviral plasmid), and lane 4 (non-template control). Bands were of the expected size at 1792 bp, indicating presence of *CAR* gene distributed between the forward and reverse primers, as shown in the schematic diagram of PCR products. (B) Vector map diagram for CS-GS397J-Lv183 lentiviral transfer plasmid encoding CAR and green fluorescent protein (GFP). The HIV-based lentiviral vector used is a third-generation lentiviral vector containing cis-active sequences of HIV-1 that are required for encapsulation of viral vector genome and transduction of target cells, including GFP, promoted by the human cytomegalovirus (CMV) promoter. The RSV promoter that drives production and expression of viral RNA in the packaging cells is located upstream of the 5′ LTR. U3 enhancer sequences, in the region of the 3′ LTR, are deleted to ensure self-inactivation of the lentiviral construct after transduction and integration into genomic DNA of target cells. [C (A, B)] Phase contrast and fluorescence image (GFP) of 293FT cells before transfection with CAR lentiviral plasmid. No GFP expression or syncytia (giant multinucleated cells) formations, which are parameters for successful lentiviral production, can be observed.[C (C, D)] Phase contrast and fluorescence image (GFP) of 293FT cells after transfection with the CAR lentiviral plasmid. Red arrows indicate syncytia (giant multinucleated cells) formation corresponding to GFP expression. GFP expression was visualized by inverted fluorescence microscopy. Cells were imaged at × 100 magnification (scale bar indicated at the bottom right corner of the image represents 200 µm).

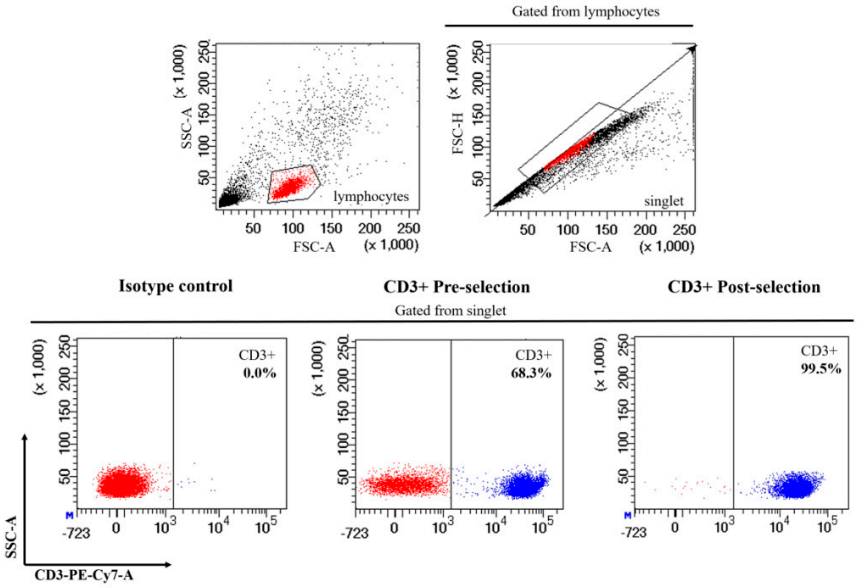

Figure S2. Immunophenotyping of peripheral blood mononucleocytes pre- and post- CD3+ T-cell magnetic selection. Peripheral blood mononucleocytes were characterized for T-cell surface marker, CD3+, immediately after isolation of the cells. Cell population was gated at lymphocytes. Singlet was gated from the lymphocyte population to remove residual cell clumps following disaggregation and eliminate auto fluorescence. Subsequently, CD3+ cells were gated from the singlet population. Results of flow cytometry show an increase in the CD3+ lymphocyte percentage from 68.3% before selection by magnetic beads to 99.5% after selection. CD3 was stained by tandem flourochrome PE-Cy 7. For each antibody, isotype-matched mouse immunoglobulin γ antibody was used as the isotype control.

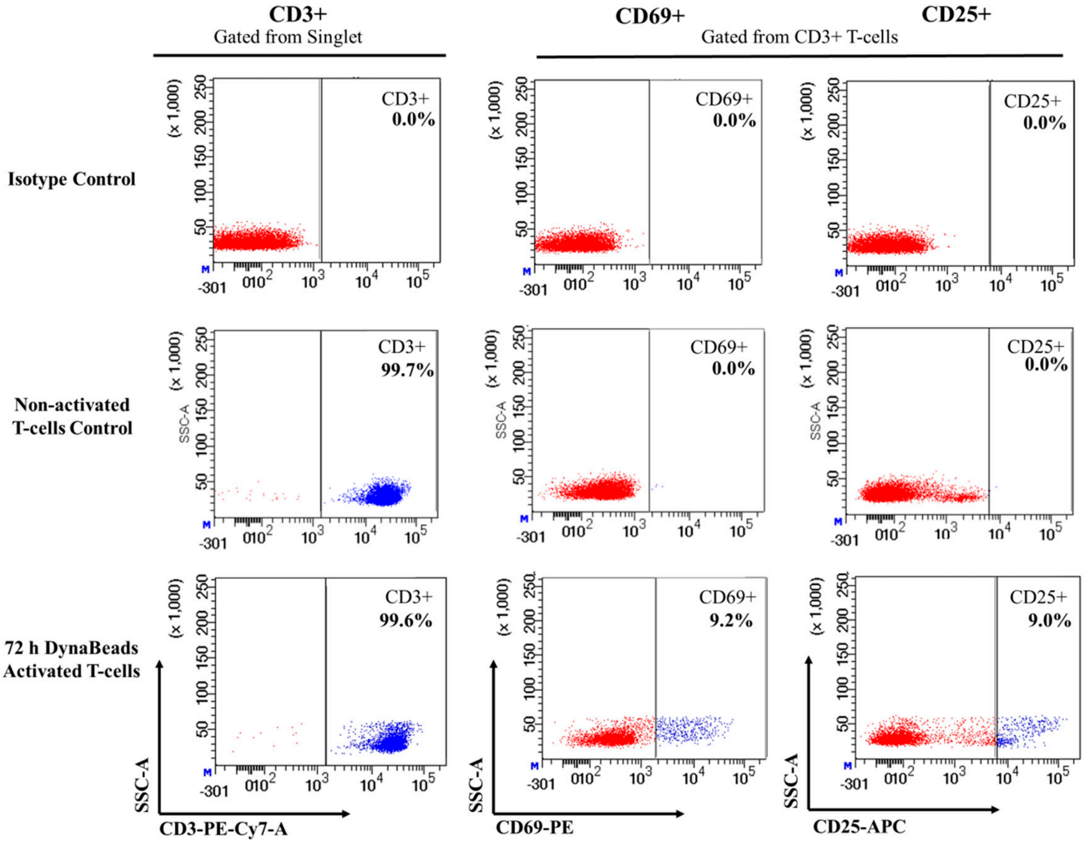

Figure S3. Immunophenotyping of DynaBead-activated T-cells for 72 h to observe background measurements of the T-cell activation markers, CD69 and CD25. Cell population was gated at lymphocytes. Singlet was gated from the lymphocyte population to remove residual cell clumps following disaggregation and eliminate auto fluorescence. Subsequently, CD3+ cells were gated from the singlet population, and CD69+ and CD25+ cells were gated from the CD3+ population. Results of flow cytometry reveal 9.2% CD69+ cells and 9.0% CD25+ cells after 72 h of activation by DynaBeads compared to non-activated T-cells. CD3 was stained by tandem flourochrome PE-Cy7-A, CD69 by PE, and CD25 by APC flourochromes. For each antibody, isotype-matched mouse immunoglobulin γ antibody was used as the isotype control.
